# Pathological remodeling and functional evolution of the corneal nervous system in ocular surface dyshomeostasis

**DOI:** 10.3389/fnagi.2026.1946017

**Published:** 2026-10-14

**Authors:** Jiayang Liu, Hanwen Lu, Xiyao Liu

**Affiliations:** 1 Department of Ophthalmology, First Affiliated Hospital of Xiamen University, School of Medicine, Xiamen University, Xiamen, Fujian, China; 2 Department of Neurosurgery and Department of Neuroscience, Fujian Key Laboratory of Brain Tumors Diagnosis and Precision Treatment, Xiamen Key Laboratory of Brain Center, the First Affiliated Hospital of Xiamen University, School of Medicine, Xiamen University, Xiamen, Fujian, China; 3 Department of Neurosurgery, First Affiliated Hospital of Xiamen University, School of Medicine, Xiamen University, Xiamen, Fujian, China

**Keywords:** aging, central sensitization, corneal esthesiometry, corneal nerve plexus, dry eye disease, *in vivo* confocal microscopy, neurodegeneration, neuroimmune signaling

## Abstract

The corneal nerve plexus is essential to ocular surface homeostasis through sensory detection, trophic support, epithelial repair, blinking, and lacrimal reflexes. In dry eye disease (DED), chronic hyperosmolar, inflammatory, mechanical, and age-related stressors alter nerve morphology and function, contributing to reduced nerve density, aberrant regeneration, microneuroma formation, sensory loss, and peripheral or central sensitization. This review summarizes the ultrastructure of corneal innervation and the molecular basis of corneal sensory transduction, with emphasis on TRPM8, TRPV1, TRPA1, Piezo2, and acid-sensing ion channels. It then examines bidirectional neuroimmune signaling involving dendritic cells, cytokines, neuropeptides, and diminished neurotrophic support. We further discuss the relationship between age-related corneal hypoesthesia, local small-fiber degeneration, and systemic neurodegenerative disorders, while noting the limited disease specificity of currently proposed corneal biomarkers. Finally, we evaluate *in vivo* confocal microscopy (IVCM) and multimodal corneal esthesiometry for structural and functional phenotyping and review emerging nerve-directed interventions. Integrating morphology, sensory function, and neuroimmune biology may improve the mechanistic classification of DED and support more precise treatment of patients with symptom–sign discordance.

## 1 Introduction

The cornea is one of the most densely innervated tissues in the human body; its neural network provides highly acute sensory perception and helps regulate ocular surface homeostasis (Al-Aqaba et al., 2019; Shaheen et al., 2014). Over the past few decades, the understanding of ocular surface diseases has undergone a major shift. Dry eye disease (DED) illustrates this evolution: early models focused predominantly on aqueous tear deficiency or isolated ocular surface inflammation (Lemp, 1995; Lemp and Foulks, 2007). However, the common clinical discordance between symptom severity and objective signs suggests that the traditional “aqueous-inflammatory” model does not fully explain the complexity of the disease. In 2017, the TFOS DEWS II report explicitly incorporated neurosensory abnormalities into the definition of DED and recognized their etiological role (Craig et al., 2017; Belmonte et al., 2017); this perspective was subsequently reaffirmed by the Asia Dry Eye Society (ADES) (Tsubota et al., 2017). This shift in consensus has directed greater attention toward pathological remodeling of the corneal nervous system in ocular surface disease.

Modern lifestyles and systemic physiological changes present persistent challenges to the structure and function of corneal nerves. For instance, altered blink patterns during prolonged visual display terminal (VDT) use can cause recurrent local hyperosmolarity and biomechanical stress on the ocular surface, subjecting superficial corneal nerve terminals to chronic physicochemical stress (Cardona et al., 2011; Himebaugh et al., 2009; Nakamura, 2018; Willcox et al., 2017). Epidemiological evidence shows that DED is more common with aging and among women, while age- and hormone-related changes may reduce neurotrophic support and the regenerative capacity of corneal nerves (Galletti and de Paiva, 2021; Schaumberg et al., 2003; Farrand et al., 2017; Stapleton et al., 2017; Uchino et al., 2011; Jie et al., 2009; Tan et al., 2015; Sullivan et al., 2017). Systemic factors such as sleep deprivation, together with central neuroplastic changes associated with chronic DED, may also disrupt peripheral sensory feedback (Li et al., 2018; Fakih et al., 2019). The effects of these environmental and systemic factors may converge on the corneal nervous system, contributing to structural remodeling and functional impairment.

Although neural mechanisms are now recognized as integral to ocular surface homeostasis, many reviews still treat nerve injury mainly as a downstream consequence of tear-film instability or inflammation. A unified neuropathological framework linking microscopic nerve remodeling, neuroimmune crosstalk, altered sensory processing, aging, and multimodal clinical assessment remains incomplete. This gap limits mechanistic interpretation of symptom–sign discordance and constrains the development of interventions guided by neural phenotyping.

To address these gaps, this review uses DED as the principal disease model to examine pathological remodeling and functional change across the corneal nervous system. We integrate basic and clinical evidence on sensory transduction, age-related neurodegeneration, neuroimmune signaling, structural and functional assessment, and nerve-directed therapy, with the aim of defining clinically relevant neural phenotypes and identifying priorities for future research.

## 2 Anatomy and physiology of the corneal nerve plexus and ocular surface homeostasis

### 2.1 Ultrastructural features of trigeminal innervation

The cornea is one of the most densely innervated tissues in the human body, with its sensory innervation derived primarily from the long ciliary nerves of the ophthalmic branch (V1) of the trigeminal nerve (Shaheen et al., 2014). These nerve fibers enter the cornea through the mid-to-posterior stroma at the limbus, extend radially toward the center and lose their myelin sheaths to form a highly complex neural network (Müller et al., 2003; Guerrero-Moreno et al., 2020). Based on anatomical stratification, corneal nerves are predominantly distributed across the stroma, Bowman’s layer, and the epithelium. Notably, the nerve fibers that penetrate Bowman’s layer to enter the basal epithelium form the subbasal nerve plexus (SBNP), which represents the densest and most physiologically active component of corneal innervation (Cruzat et al., 2017; Marfurt et al., 2010).

At the ultrastructural level, the SBNP is composed of numerous parallel nerve fiber bundles, exhibiting a characteristic whorl-like distribution pattern centered around the central corneal whorl (Patel and McGhee, 2005; Feng et al., 2025). Studies have demonstrated that the nerve density of the human SBNP is influenced by age, anatomical location, and systemic physiological factors (Parissi et al., 2013). The subbasal nerves give rise to vertical branches that course between epithelial cells, forming intraepithelial nerve terminals that interface directly with the external environment and transduce various physical and chemical stimuli (Belmonte et al., 2017; Müller et al., 2003). This unique anatomical configuration endows the cornea with high sensitivity to changes in the ocular surface microenvironment, providing the structural basis for maintaining physiological homeostasis (Figure 1).

**Figure 1:**
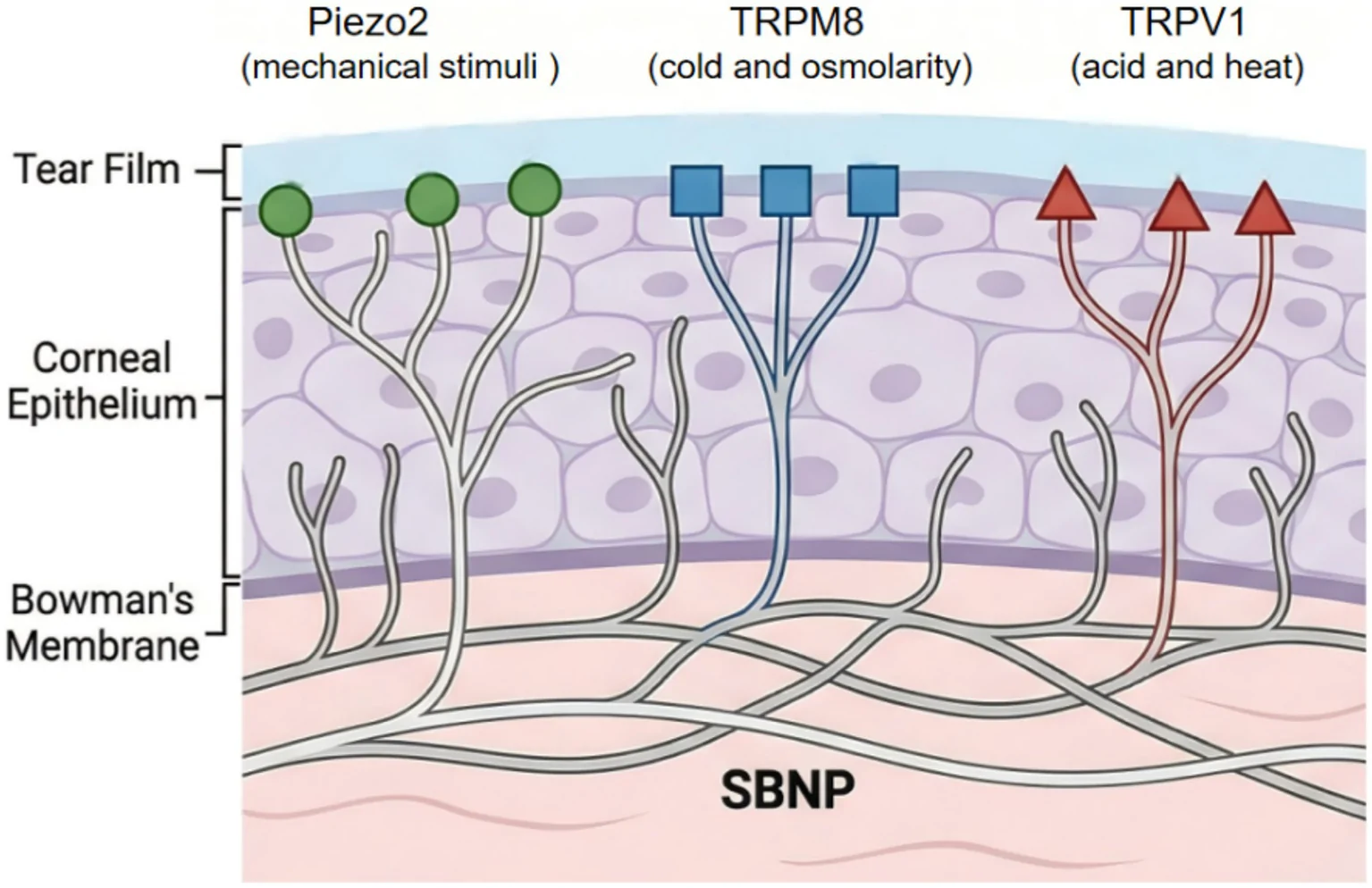
Corneal nerve fibers penetrate upward from the stroma through Bowman’s layer to form the dense subbasal nerve plexus (SBNP) and further branch into intraepithelial nerve terminals. Specific ion channels are expressed on the cell membranes of these nerve terminals to sense the ocular surface microenvironment: TRPM8 (blue) detects temperature drops and tear hyperosmolar stress; Piezo2 (green) monitors changes in mechanical tension, such as blinking; and TRPV1 (red) sense acidic microenvironments and inflammatory pain stimuli.

Protection and regeneration of the corneal nerve network after injury are also relevant to neurorestoratology (Huang et al., 2021). Evidence from noncorneal neural-repair models suggests that oxidative stress and the local microenvironment influence neuronal survival; however, extrapolation of findings from neural stem-cell transplantation to corneal nerve repair remains speculative and requires direct experimental validation (Gao et al., 2013).

### 2.2 The afferent-efferent neural homeostatic network of the lacrimal functional unit (LFU)

The lacrimal functional unit (LFU) is an integrated regulatory system comprising the cornea, conjunctiva, accessory lacrimal glands, meibomian glands, and their interconnecting neural pathways. It maintains ocular surface homeostasis through coordinated regulation of the tear film (Willcox et al., 2017; Dartt, 2009). Corneal sensory nerves act as the afferent limb of this system by detecting changes in the ocular surface microenvironment and initiating compensatory responses (Belmonte et al., 2017). Sensory input travels through the ophthalmic division of the trigeminal nerve to lacrimal secretomotor centers in the brainstem, which activate parasympathetic efferents to the lacrimal glands (Meng and Kurose, 2013). Different corneal sensory-neuron subtypes contribute differently to tear secretion.

Basal tear secretion is regulated by low-threshold cold thermoreceptors. When tear film breakup causes a localized temperature drop or aqueous evaporation induces mild hyperosmolarity, the background discharge activity maintained by these cold receptors sustains basal tear flow via low-threshold reflex loops (Parra et al., 2010; Quallo et al., 2015). Reflex tear secretion is predominantly mediated by polymodal nociceptors. Upon exposure to chemical stimuli, mechanical injury, or significant hyperosmolar stress, these high-threshold fibers are activated, triggering reflex tearing to wash away irritants and protect the ocular surface epithelium (Belmonte et al., 2017; Meng and Kurose, 2013).

The blink reflex serves as another important neural mechanism for maintaining ocular surface homeostasis. The corneal subbasal nerve plexus not only conducts tactile and nociceptive sensations but also modulates blink dynamics by monitoring changes in the physical parameters of the ocular surface (Kaminer et al., 2011). Within the neural drive mechanism of spontaneous blinking, ocular surface nerve terminals actively monitor tear film breakup. When the loss of tear film continuity leads to the direct exposure of the corneal epithelium to air, the resultant localized biomechanical strain and hyperosmolar signals elicit the blink reflex via trigeminal afferent pathways (Quallo et al., 2015; Hirata et al., 2018). Furthermore, neurally mediated blinking not only facilitates the even distribution of tears across the ocular surface but also promotes lipid release through the mechanical compression of the meibomian glands by the orbicularis oculi muscle, thereby enhancing tear film stability (Willcox et al., 2017; Rodriguez et al., 2018).

Under conditions of corneal neural dyshomeostasis, this precise coupling is often disrupted due to altered neural sensitivity or impaired feedback loops. For instance, during prolonged use of visual display terminals (VDTs), sustained visual attention reduces the blink rate. This suppression further exacerbates the neural stress induced by tear film breakup, thereby contributing to a vicious cycle of ocular surface damage (Cardona et al., 2011; Himebaugh et al., 2009; Nakamura, 2018) (Figure 2).

**Figure 2:**
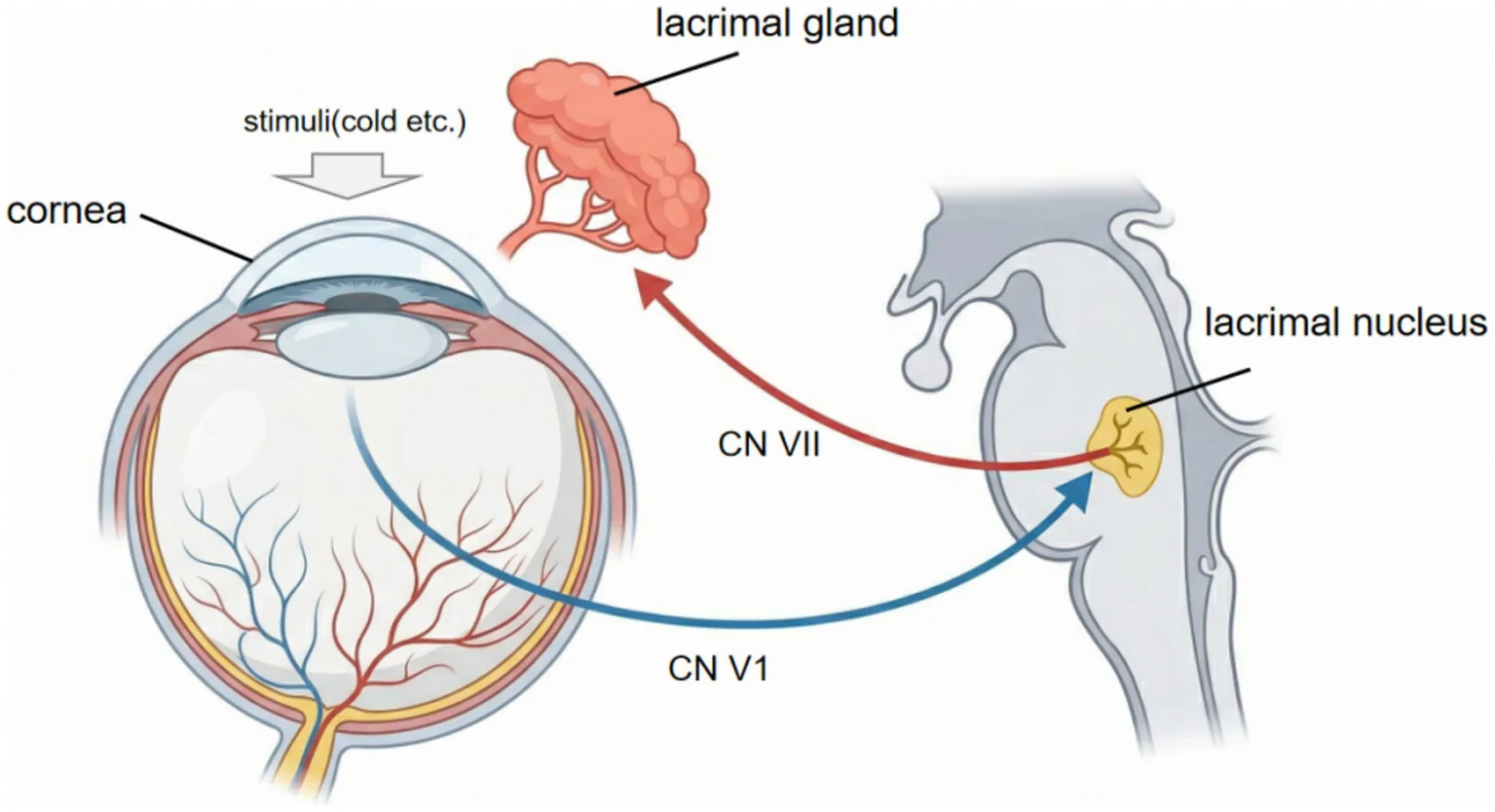
Neural signals are transmitted via the trigeminal afferent pathway (Cranial Nerve V1, CN V1), converging at the trigeminal ganglion and ascending to the brainstem centers. Following signal integration in the brainstem, feedback regulation is conducted through Cranial Nerve VII (CN VII), thereby maintaining the physiological homeostasis of the tear film and the ocular surface microenvironment.

### 2.3 Molecular transduction basis of sensory receptors

The high sensitivity of corneal nerve terminals to physical and chemical stimuli depends on a diverse array of specific ion channels and receptors expressed on their cell membranes. These molecular elements convert environmental signals at the ocular surface into electrical impulses, constituting the molecular foundation for maintaining ocular surface homeostasis.

In terms of mechanosensation, the expression of Piezo2 in corneal afferent neurons plays an important role. As a low-threshold mechanosensitive ion channel, Piezo2 rapidly opens in response to minute changes in cell membrane tension, triggering cation influx and generating action potentials. This mechanosensory feedback mechanism not only mediates corneal tactile perception but is also crucial for monitoring dynamic changes in tear film thickness and maintaining a normal spontaneous blink rate (Fernández-Trillo et al., 2020; Bron et al., 2014).

Cold thermosensation and osmolarity surveillance are primarily mediated by the transient receptor potential melastatin 8 (TRPM8) channel. Serving as a key molecular marker for corneal cold thermoreceptors, TRPM8 exhibits high sensitivity to environmental temperature drops and hyperosmolar stress induced by tear evaporation (Parra et al., 2010; Alcalde et al., 2018). Under physiological conditions, the background discharge activity mediated by TRPM8 is the primary driving force for sustaining basal tear secretion. Conversely, in pathological processes such as DED, overactivation or altered expression of TRPM8 channels may contribute to the characteristic sensations of dryness and abnormal cooling experienced by patients (Parra et al., 2010; Quallo et al., 2015).

Nociceptive signaling of potential tissue damage is co-mediated by the transient receptor potential vanilloid 1 (TRPV1) and transient receptor potential ankyrin 1 (TRPA1) channels expressed on polymodal nociceptors (Bautista et al., 2006; Alamri et al., 2015; Schecterson et al., 2020). TRPV1 responds to noxious heat, acidic microenvironments, and severe tear hyperosmolarity, and can promote DED-associated inflammatory cascades and neurogenic pain transmission (Immke and Gavva, 2006; Murata and Masuko, 2006; Pan et al., 2011). Concurrently, acid-sensing ion channels (ASICs) participate in monitoring fluctuations in ocular surface pH, functioning synergistically with the aforementioned TRP channels to support sensory appraisal in the cornea (Callejo et al., 2015). The integrated activity of these molecular channels enables the cornea to detect a broad range of stimuli, ranging from innocuous mechanical perturbations to severe pathological damage (Table 1).

**Table 1:** Summary of the roles of corneal sensory receptors and ion channels in dry eye disease.

Receptor/ion channel	Receptor type	Physiological activation and regulatory function	Pathological role and clinical manifestation in DED
Piezo2	Low-threshold mechanoreceptor	Activation mechanism: Senses minute changes in cell membrane tension (e.g., touch). Physiological function: Monitors dynamic changes in tear film thickness and maintains normal spontaneous blink frequency (Fernández-Trillo et al., 2020; Bron et al., 2014).	Abnormal mechanical sensation may impair the blink feedback mechanism, exacerbating the vicious cycle of tear film breakup and ocular surface exposure (Fernández-Trillo et al., 2020; Bron et al., 2014).
TRPM8	Cold thermoreceptor	Activation mechanism: Detects environmental temperature drops and hyperosmolar stress caused by tear evaporation. Physiological function: Maintains normal basal tear secretion through background discharge activity (Parra et al., 2010; Alcalde et al., 2018).	Overactivation or abnormal expression of the channel is a potential molecular contributor to the typical dryness and abnormal cold sensations in DED patients (Parra et al., 2010; Quallo et al., 2015).
TRPV1	Polymodal nociceptor	Activation mechanism: Responds to noxious heat, acidic microenvironments, and severe tear hyperosmolarity. Physiological function: Mediates reflex tearing to wash away irritants (Alamri et al., 2015; Immke and Gavva, 2006).	Persistent activation and sensitization of the channel can promote local inflammatory responses on the ocular surface and mediate neurogenic pain conduction (hyperalgesia) (Murata and Masuko, 2006; Pan et al., 2011).
TRPA1	Polymodal nociceptor	Activation mechanism: Responds to extreme cold, exogenous chemical irritants, and endogenous inflammatory mediators. Physiological function: Synergizes with TRPV1 to detect potential tissue-damage signals (Bautista et al., 2006; Schecterson et al., 2020).	Synergizes with TRPV1 under chronic inflammation and hyperosmolar stress to further amplify nociceptive signal transduction in peripheral nerves (Bautista et al., 2006; Schecterson et al., 2020).
ASICs	Acid-sensing ion channels	Activation mechanism: Monitors fluctuations in the pH of the ocular surface microenvironment. Physiological function: Participates in the precise sensory evaluation system of the cornea (Callejo et al., 2015).	Activated in the DED-associated inflammatory acidic microenvironment, synergizing with the TRP family to increase nerve-terminal hypersensitivity (Callejo et al., 2015).

## 3 Pathomorphology of corneal nerves in dry eye disease

### 3.1 Morphological manifestations of neurodegenerative damage and the pathological significance of microneuromas

In the context of DED-associated corneal nerve damage, the formation of microneuromas is an important morphological feature of aberrant nerve terminal regeneration. Observed via *in vivo* confocal microscopy (IVCM), microneuromas present as abnormal spherical or bulbous hyper-reflective structures at the terminal or mid-portions of the subbasal nerve plexus. These structures are thought to represent the disorganized accumulation and aberrant encapsulation of damaged nerve axons attempting to re-innervate the epithelium (Dermer et al., 2022; Chinnery et al., 2022). Studies have reported that the detection rate of these pathological alterations is significantly higher in DED patients compared to healthy controls, and they are particularly prevalent in patients presenting with severe neuropathic pain (Moein et al., 2020).

Microneuromas may represent both structural abnormalities and a substrate for altered nerve function. With altered ion-channel expression and membrane excitability, these structures may exhibit ectopic spontaneous activity and heightened responses to mechanical and chemical stimuli (Belmonte et al., 2017; Costigan et al., 2009). Clinical studies have reported an association between the presence of corneal microneuromas and patients’ subjective pain scores. Particularly in patients with minimal clinical signs but extreme pain sensitivity, microneuromas are considered a potential marker of and contributor to peripheral sensitization (Belmonte et al., 2017; Dermer et al., 2022). Furthermore, the development of microneuromas may reflect the chronic inflammatory state of the ocular surface microenvironment. When the ocular surface is chronically exposed to hyperosmolarity and pro-inflammatory cytokines, the nerve repair process is disrupted, preventing nerve terminals from forming organized terminal arborization and potentially creating sites of persistent ectopic activity (Moein et al., 2020; Goyal and Hamrah, 2016).

### 3.2 Modulatory effects of age and sex on corneal nerve remodeling

Age is a fundamental factor influencing the anatomical structure and physiological function of corneal nerves. Clinical studies reveal that with advancing age, healthy populations exhibit a physiological decline in subbasal nerve plexus density, accompanied by a gradual increase in nerve tortuosity (Parissi et al., 2013; Gambato et al., 2015). In the context of DED, neurodegenerative changes are more pronounced in older patients, manifesting as diminished repair capacity and reduced neurotrophic support (Vereertbrugghen and Galletti, 2022; Lambiase et al., 2000). Moreover, older patients typically experience a reduction in corneal sensation; this sensory blunting can mask the severity of ocular surface damage, thereby exacerbating the discrepancy between clinical signs and subjective symptoms (Belmonte et al., 2017; Tsubota et al., 2017).

Reduced corneal sensation and neurodegenerative change may form a bidirectional relationship. Loss of subbasal nerve fibers, terminal retraction, or sensory-receptor dysfunction can raise corneal sensory thresholds; conversely, diminished afferent drive can weaken reflex blinking and lacrimal secretion, destabilize the tear film, impair epithelial repair, and reduce local trophic support, thereby promoting further nerve degeneration (Dartt, 2009; Meng and Kurose, 2013; Lambiase et al., 2000; Labbé et al., 2012; Labbé et al., 2013). In the broader context of aging neuroscience, corneal small-fiber loss has also been reported in individuals with mild cognitive impairment who subsequently progressed to dementia and in patients with Parkinson’s disease or multiple system atrophy (Ponirakis et al., 2023; Niu et al., 2024). These observations suggest that combining corneal esthesiometry with IVCM-derived structural measures may provide a candidate window into peripheral small-fiber injury and systemic neurodegenerative processes. However, corneal sensation, nerve density, and central neurodegeneration do not show a simple one-to-one relationship and are influenced by age, diabetes, ocular surface inflammation, medication use, and other confounders; these measures should therefore not yet be regarded as disease-specific diagnostic biomarkers.

Sex differences also play a pivotal role in corneal nerve remodeling and DED pathogenesis. Epidemiological data show that both the prevalence and symptom severity of DED are generally higher in women than in men (Schaumberg et al., 2003). From a neurobiological perspective, fluctuations in sex hormone levels (e.g., estrogens and androgens) can influence the sensitivity and regenerative capacity of corneal nerves. Alterations in estrogen levels are believed to be associated with enhanced ocular surface inflammatory responses and the sensitization of sensory nerves, which may contribute to why perimenopausal women are more susceptible to pronounced ocular discomfort and neuropathic pain (Sullivan et al., 2017; Goyal and Hamrah, 2016). In contrast, androgens may have protective effects on lacrimal gland function and nerve fibers (Dartt, 2009; Bron et al., 2017). Together, these biological differences contribute to heterogeneity between sexes regarding corneal nerve damage patterns and pain perception (Table 2).

**Table 2:** Morphological changes of corneal nerves associated with dry eye disease and their clinical significance.

Morphological abnormality	Microstructural features	Clinical and pathological significance
Decreased density and fragmentation	The total density of the subbasal nerve plexus decreases significantly, with reduced branching and fragmentation of main nerve trunks (Alhatem et al., 2012; Labbé et al., 2012; Labbé et al., 2013). It exhibits spatial heterogeneity (often spreading peripherally from the center or tear film breakup areas) (Patel and McGhee, 2005; Efraim et al., 2020).	The degree of reduction correlates with ocular surface staining and decreased tearing; it impairs corneal sensory perception to external stimuli; the reduced release of local neurotrophic factors further exacerbates epithelial repair defects (Lambiase et al., 2000; Labbé et al., 2013; Labetoulle et al., 2019).
Increased nerve fiber tortuosity	Pathological remodeling of nerve fiber pathways, manifesting as curling or irregular bending (Cruzat et al., 2017; Labbé et al., 2013).	Reflects abnormal regenerative attempts of nerve fibers in a damaging environment of chronic inflammation and hyperosmolar stress, marking the breakdown of local microenvironmental homeostasis (Cruzat et al., 2017; Vereertbrugghen and Galletti, 2022).
Microneuroma formation	Abnormal globular or bulbous hyperreflective structures at the nerve terminals or mid-segments; essentially the disorganized accumulation and abnormal encapsulation of damaged nerve axons (Dermer et al., 2022; Chinnery et al., 2022).	Altered ion-channel expression and membrane excitability may promote ectopic spontaneous activity and hypersensitivity (Belmonte et al., 2017; Costigan et al., 2009); positively correlated with subjective pain scores, and may serve as structural markers of peripheral sensitization and the symptom-sign mismatch (Belmonte et al., 2017; Dermer et al., 2022; Moein et al., 2020).
Age-related neurodegeneration	Older patients not only present with physiological density decline and increased tortuosity (Parissi et al., 2013; Gambato et al., 2015), but also a significant lack of pathological repair capacity and neurotrophic factor support (Vereertbrugghen and Galletti, 2022; Lambiase et al., 2000).	Frequently leads to decreased corneal sensation; this sensory blunting may mask the true severity of ocular surface damage, further exacerbating the discrepancy between clinical signs and subjective symptoms (Belmonte et al., 2017; Tsubota et al., 2017).
Hormone/sex-related remodeling heterogeneity	The sensitivity and regenerative capacity of corneal nerves fluctuate with changes in sex hormone levels (estrogens and androgens) (Sullivan et al., 2017).	Estrogen alterations (e.g., during perimenopause) tend to enhance ocular surface inflammation and sensory nerve sensitization, inducing pronounced discomfort and neuropathic pain (Sullivan et al., 2017; Goyal and Hamrah, 2016); androgens exert a protective role, contributing to sex-related heterogeneity in DED manifestations (Dartt, 2009; Bron et al., 2017).

## 4 The neuro-immune-inflammatory axis a central mechanism of DED-associated corneal nerve damage

### 4.1 Anatomical coupling between corneal nerves and resident immune cells

The cornea is not only one of the most densely innervated tissues in the body but also contains a network of resident immune cells, among which bone marrow-derived dendritic cells (DCs) are prominent. Under physiological conditions, these immune cells exhibit close spatial coupling with the subbasal nerve plexus (Chinnery et al., 2017; Hamrah et al., 2003). Studies have demonstrated that DCs in the basal epithelium are frequently distributed along nerve fibers, forming direct physical contacts with them—a structural arrangement termed the “neuro-immune unit” (Wu et al., 2022; Hamrah and Dana, 2007).

In the pathological setting of DED, this homeostatic coupling undergoes significant alterations. Concomitant with nerve fiber degeneration, the density of DCs in both the central and peripheral cornea increases significantly, and their cellular morphology transitions from a resting state (round and clustered) to an activated state (dendritiform with long processes) (Kheirkhah et al., 2015; Uchino et al., 2023; Yamaguchi et al., 2016). Activated DCs secrete a variety of pro-inflammatory cytokines, such as interleukin-1 (IL-1) and tumor necrosis factor-α (TNF-α). These cytokines act directly on receptors located on nerve terminals, lowering their activation thresholds and thereby inducing peripheral sensitization (Pan et al., 2011; Vereertbrugghen and Galletti, 2022). Concurrently, damaged nerve terminals in turn modulate the recruitment and maturation of immune cells by releasing neuropeptides, such as substance P (SP) and calcitonin gene-related peptide (CGRP) (Murata and Masuko, 2006; Labetoulle et al., 2019). This bidirectional crosstalk between the neural and immune systems constitutes an important mechanism for the chronicity and persistence of DED-associated inflammation.

### 4.2 The bidirectional regulatory loop of neuropeptides and pro-inflammatory cytokines

In the pathogenesis of DED, neurogenic inflammation forms a self-sustaining vicious cycle through the complex interplay between neuropeptides and immune mediators. When the cornea is damaged or exposed to a hyperosmolar microenvironment, sensory nerve terminals release key neuropeptides, including SP and CGRP (Murata and Masuko, 2006; Sabatino et al., 2017). In limbal and adjacent vascular tissues, SP can induce vasodilation, increase vascular permeability, and directly promote the recruitment of DCs and macrophages to the inflammatory site. Furthermore, it upregulates their capacity to express major histocompatibility complex class II (MHC-II) molecules, thereby enhancing antigen presentation (Chinnery et al., 2017; Hamrah and Dana, 2007).

Simultaneously, pro-inflammatory cytokines (such as IL-1β, TNF-α, and IL-6) produced by activated immune cells act on nearby nerve terminals. These inflammatory mediators directly sensitize nociceptive channels, such as TRPV1, significantly lowering the mechanical and thermal stimulation thresholds of nerve terminals (Pan et al., 2011; Vereertbrugghen and Galletti, 2022). Evidence suggests that chronic exposure to these pro-inflammatory cytokines leads to alterations in intracellular signaling pathways within nerve terminals, triggering terminal retraction and aberrant regeneration (e.g., the formation of microneuromas), which subsequently release even more neuropeptides (Cruzat et al., 2017; Guzmán et al., 2020). This bidirectional feedback loop not only explains the parallel manifestation of ocular surface damage and hyperalgesia in DED but also provides a biological basis for the transition of the disease from acute inflammation to a chronic, protracted state (Costigan et al., 2009; Goyal and Hamrah, 2016).

Persistent nociceptive input from the cornea is conveyed through the ophthalmic division of the trigeminal nerve to the trigeminal ganglion and then to the trigeminal sensory complex in the brainstem. Repeated input can increase the excitability and synaptic efficacy of second-order neurons and engage glia-dependent plasticity, thereby establishing central sensitization (Fakih et al., 2019; Guerrero-Moreno et al., 2020; Costigan et al., 2009; Goyal and Hamrah, 2016). Consequently, amplified central processing may sustain spontaneous pain, hyperalgesia, or allodynia even after peripheral ocular surface injury has become mild or partially resolved, providing a mechanistic explanation for symptom–sign discordance in some patients with DED (Guerrero-Moreno et al., 2020; Goyal and Hamrah, 2016; Galor et al., 2018).

### 4.3 Depletion mechanisms of neurotrophic support under chronic stress

Under physiological conditions, a tight, mutually trophic relationship exists between corneal epithelial cells and nerve terminals. Nerve growth factor (NGF), acting as the core molecule in this system, not only maintains the survival and axonal integrity of sensory nerves but also safeguards corneal barrier function by promoting the proliferation and migration of epithelial cells (Lambiase et al., 2000; Sacchetti and Lambiase, 2017). However, during the chronic pathological progression of DED, this neurotrophic support system frequently becomes dysfunctional (Lambiase et al., 2000).

Studies indicate that prolonged exposure to ocular surface hyperosmolarity and pro-inflammatory cytokines can disrupt local NGF synthesis, secretion, and signaling (Vereertbrugghen and Galletti, 2022; Sacchetti and Lambiase, 2017). When the local concentration of neurotrophic factors becomes insufficient to maintain homeostasis, corneal nerves exhibit pronounced neurodegenerative changes, including decreased plexus density and terminal retraction. This, in turn, attenuates the neural regulation of epithelial repair, leading to recurrent damage to the corneal epithelium (Lambiase et al., 2000; Labbé et al., 2013). Furthermore, chronic stress may lead to the downregulation or altered sensitivity of NGF receptors (such as TrkA) on nerve terminals, reducing the biological effects of residual trophic factors (Müller et al., 2003; Sacchetti and Lambiase, 2017). Disruption of this mutually trophic relationship may mark an important transition, signifying the transition of the ocular surface from reversible inflammatory damage to chronic neurodegenerative pathology (Goyal and Hamrah, 2016; Sacchetti and Lambiase, 2017).

## 5 Clinical diagnosis and assessment techniques

### 5.1 Application of *in vivo* confocal microscopy in neuroimaging

*In vivo* confocal microscopy (IVCM) is the principal clinical modality capable of real-time, non-invasive imaging of corneal nerves at cellular resolution. Through IVCM, clinicians can directly observe and quantify the morphological parameters of the subbasal nerve plexus (SBNP), including nerve density, tortuosity, branching, and the presence of microneuromas (Cruzat et al., 2017; Moein et al., 2020). Studies have reported associations between reduced SBNP density and subjective symptoms in DED, although the strength of these associations varies. This makes IVCM a valuable *in vivo* imaging tool for assessing corneal neuromorphological abnormalities, although its standardization and precise correlation with functional deficits remain to be optimized (Labbé et al., 2012; Labbé et al., 2013).

IVCM can also characterize interactions within the neuroimmune unit. Quantitative and morphological analysis of central corneal dendritic cells may provide information on ocular surface inflammatory activity and its spatial relationship to nerve fibers (Kheirkhah et al., 2015; Yamaguchi et al., 2016). These measures may complement clinical assessment and may help monitor responses to neuroregenerative interventions, including recombinant human nerve growth factor, but they are not yet sufficiently standardized for stand-alone diagnosis or treatment selection (Sacchetti and Lambiase, 2017; Bonini et al., 2018).

### 5.2 Corneal esthesiometry and functional assessment

A commonly used method for evaluating corneal nerve function is corneal esthesiometry. The traditional Cochet-Bonnet esthesiometer determines the corneal mechanical sensitivity threshold by adjusting the length of a nylon monofilament (Belmonte et al., 2017; Belmonte et al., 1999). Although simple to operate, this method primarily evaluates the function of mechanosensitive nociceptors and has limited precision in assessing hypersensitive states (hyperalgesia) (Belmonte et al., 2017; Goyal and Hamrah, 2016).

To more comprehensively evaluate nerve function in DED patients, researchers have developed the non-contact corneal esthesiometer (NCCE). By controlling the temperature, pressure, and chemical composition (e.g., CO₂ concentration) of an air pulse, this device can assess the reactivity of cold thermoreceptors, mechanoreceptors, and chemoreceptors (Belmonte et al., 2017; Belmonte et al., 1999). Such multimodal functional assessment assists in distinguishing between peripheral sensory loss and abnormal pain perception driven by central sensitization, providing a functional basis for personalized DED treatment regimens—particularly interventions targeting neuropathic pain (Goyal and Hamrah, 2016; Galor et al., 2018).

## 6 Therapeutic strategies and future perspectives

### 6.1 Interventional strategies targeting neuroprotection and regeneration

Given the neurodegenerative features observed in DED, clinical management is expanding beyond tear replacement to include strategies that support neural repair. Nerve growth factor (NGF) is a key regulator of corneal neural homeostasis, and recombinant human NGF (e.g., cenegermin) has demonstrated clinically meaningful neuroregenerative effects in neurotrophic keratitis (Sacchetti and Lambiase, 2017; Bonini et al., 2018). These findings provide a rationale for investigating whether NGF-based therapy can restore SBNP structure, corneal sensation, and lacrimal functional unit feedback in patients with DED-associated nerve dysfunction.

Additionally, autologous serum eye drops, rich in neurotrophic factors, epidermal growth factor (EGF), and anti-inflammatory cytokines, are widely used in the treatment of severe DED accompanied by nerve damage (Matsumoto et al., 2004; Geerling et al., 2004). Serum components can simulate the physiological microenvironment of the ocular surface. By antagonizing the detrimental effects of pro-inflammatory cytokines, they provide essential metabolic support and survival signals to damaged nerves (Lambiase et al., 2000; Geerling et al., 2004). Such biological preparations may improve nerve morphology and alleviate neuropathic pain (Galor et al., 2018; Aggarwal et al., 2019).

### 6.2 Molecular targets and future research directions

As understanding of ion-channel mechanisms advances, the development of drugs targeting TRP channels has become a major research focus. TRPM8 agonists can enhance tear secretion by activating corneal cold thermoreceptors. Acoltremon ophthalmic solution 0.003% (formerly AR-15512) was approved by the US FDA in May 2025 for the treatment of the signs and symptoms of dry eye disease in adults (Parra et al., 2010; Quallo et al., 2015). Concurrently, the development of TRPV1 antagonists focuses on blocking neurogenic inflammation and mitigating chronic pain, offering novel analgesic strategies for patients with severe symptoms but minimal signs (Immke and Gavva, 2006; Murata and Masuko, 2006; Pan et al., 2011).

In future work, clinical research and management should place greater emphasis on the integration of the neuro-immune dimension. Utilizing artificial intelligence (AI)-assisted automated quantification of IVCM images, combined with molecular biological approaches for precise profiling of the ocular surface microenvironment, may support individualized neuroprotective regimens (Setu et al., 2022; Wei et al., 2020). Future research priorities should focus on breaking the vicious cycle of nerve damage and inflammatory exacerbation, and on achieving long-term homeostatic restoration of corneal nerve function through local microenvironmental modulation (Al-Aqaba et al., 2019; Goyal and Hamrah, 2016) (Table 3).

**Table 3:** Mechanisms, clinical progress, and limitations of nerve-targeted therapies for dry eye disease.

Therapeutic strategy	Representative drug/preparation	Core mechanism of action	Clinical progress and current application	Limitations and challenges
Neurotrophic and regenerative	Recombinant human nerve growth factor (rhNGF, Cenegermin)	Directly binds to TrkA receptors on nerve terminals to promote the regeneration and survival of damaged nerve axons; supports the bidirectional neuroepithelial trophic feedback loop (Sacchetti and Lambiase, 2017; Bonini et al., 2018).	Approved for neurotrophic keratitis (NK); evidence for improving nerve density and sensation in DED-associated nerve damage remains preliminary (Sacchetti and Lambiase, 2017; Bonini et al., 2018).	High treatment costs; requires cold-chain storage and strict dosing frequency; transient ocular pain may occur in some patients during initial use.
Biological microenvironment substitution	Autologous Serum Eye Drops (ASEDs)	Simulates natural tears, rich in neurotrophic factors (NGF, BDNF), epidermal growth factor, and anti-inflammatory factors; provides metabolic support and antagonizes pro-inflammatory damage (Lambiase et al., 2000; Geerling et al., 2004).	Widely used as a second- or third-line treatment for severe DED accompanied by neuropathic pain or persistent epithelial defects (Matsumoto et al., 2004; Aggarwal et al., 2019).	Requires repeated blood draws with contraindications (e.g., anemia/infectious diseases); lacks unified standardized preparation protocols; potential risk of contamination and inconvenient storage.
Sensory channel modulation (Secretagogue and analgesic)	TRPM8 agonists (e.g., AR-15512)	Simulates cold sensory signals, selectively activating the TRPM8 channels on cold thermoreceptors to enhance basal tear secretion via the low-threshold reflex loop (Parra et al., 2010; Quallo et al., 2015).	Acoltremon ophthalmic solution 0.003% (formerly AR-15512; TRYPTYR) was approved by the US FDA in May 2025 for the treatment of the signs and symptoms of dry eye disease in adults. Its long-term effects on corneal nerve remodeling remain to be established.	Initial topical application may cause ocular stinging or abnormal cold sensation; the effects of long-term use on the remodeling of damaged corneal nerves remain unclear.
Neurogenic inflammation blockade	TRPV1-targeting agents (e.g., tivanisiran)	Reduces TRPV1-mediated nociceptive signaling, inhibits the abnormal release of neuropeptides, and breaks the vicious cycle of nerve damage and inflammation exacerbation (Immke and Gavva, 2006; Pan et al., 2011).	Currently in active clinical development and experimental validation, primarily targeting patients with DED-related neuropathic pain and marked symptom–sign discordance.	Excessive blockade may diminish protective corneal sensory reflexes (masking true ocular surface damage); limited efficacy for predominantly aqueous-deficient DED (Goyal and Hamrah, 2016).
